# Understanding others’ preferences: A comparison across primate species and human societies

**DOI:** 10.1371/journal.pone.0295221

**Published:** 2024-01-17

**Authors:** Juliane Kaminski, Roman Stengelin, Antje Girndt, Daniel Haun, Katja Liebal

**Affiliations:** 1 Department of Psychology, University of Portsmouth, Portsmouth, United Kingdom; 2 Department of Comparative Cultural Psychology, Max-Planck Institute for Evolutionary Anthropology, Leipzig, Germany; 3 Department of Psychology and Social Work, University of Namibia, Windhoek, Namibia; 4 Department of Developmental and Comparative Psychology, Max-Planck Institute for Evolutionary Anthropology, Leipzig, Germany; 5 Faculty of Education, Leipzig Research Centre for Early Child Development & Department for Early Child Development and Culture, Leipzig University, Leipzig, Germany; 6 Life Sciences, Institute of Biology, Leipzig University, Leipzig, Germany; University of Birmingham, UNITED KINGDOM

## Abstract

We investigated children’s and non-human great apes’ ability to anticipate others’ choices from their evident food preferences—regardless of whether these preferences deviate or align with one’s own. We assessed children from three culturally-diverse societies (Namibia, Germany, and Samoa; N = 71; age range = 5–11) and four non-human great ape species (chimpanzees (*Pan troglodytes*), bonobos (*Pan paniscus*), gorillas (*Gorilla gorilla*), and orangutans (*Pongo abelii*); N = 25; age range = 7–29) regarding their choices in a dyadic food-retrieval task. Across conditions, participants’ preferences were either aligned (*same preference* condition) or opposed (*opposite preference* condition) to those of their competitors. Children across societies altered their choices based on their competitor’s preferences, indicating a cross-culturally recurrent capacity to anticipate others’ choices relying on preferences-based inferences. In contrast to human children, all non-human great apes chose according to their own preferences but independent of those of their competitors. In sum, these results suggest that the tendency to anticipate others’ choices based on their food preferences is cross-culturally robust and, among the great apes, most likely specific to humans.

## Introduction

One key aspect of human social cognition is understanding and making inferences about others’ mental states, such as their perceptions, knowledge, beliefs, desires, and preferences by utilizing a *Theory of Mind* [1,2]. There is evidence that other primate species also understand the perceptual and knowledge states of others [3–5]. Yet, evidence in support of an explicit, deliberate understanding of others’ desires and beliefs among our phylogenetic cousins, the non-human great apes (henceforth great apes), is still sparse [4,6,7], but see [8–10]. Because of the dearth of evidence for non-human primates’ knowledge of others’ desires and beliefs, it has been suggested that such social cognitive skills are not just unique to humans, but also part of a cognitive skill set that is definitive of the human species.

The claim that a specific trait is at the core of what it means to be human implies that the trait is recurrent across different human societies. In many cognitive domains, however, humans differ substantially across populations. This includes variation in visual perception [11,12], spatial memory [13–15], numerical cognition [16,17], social learning strategies [18–22] as well as cooperation, fairness, and prosociality [23–26]. As many cognitive skills vary cross-culturally, universality in any domain, including social cognition, requires the comparison of humans from different socio-cultural contexts. Hence, systematic efforts combining cross-species, cross-cultural comparisons including both great apes and humans from different cultural contexts are essential to identify which skills are uniquely human and which are shared with other great apes [27].

In the present study, we use such a combined comparative approach to investigate a core facet of social cognition: The ability to understand and predict others’ choices utilizing their preferences, even if such preferences may differ from one’s own. From their second year of life, urban children from Western societies (i.e., US, Germany) take others’ potentially divergent preferences into account when predicting their choices [28–30]. In the classic “broccoli task”, 14- and 18-month-old children chose between a cracker and a piece of broccoli [29]. Though most children preferred the cracker, the 18-month-olds, but not the younger children, readily assigned the opposite preference to another person whom they had witnessed expressing disgust toward the cracker and excitement toward the broccoli. Thus, 18-month-old children differentiated between their own and others’ desires and related such preferences to cues indicating their partners’ emotional states [29], but see [31,32] for failed replications of this effect among older participants.

Using a storytelling paradigm, Wright Cassidy and colleagues (2005) found 3-year-old US children were able to predict an agent’s choice based on their preferences as long as the agent’s preferences did not conflict with theirs [33]. At the age of 4 years, children mastered the task regardless of whether their preferences aligned with those of the agent. Using a paradigm with reduced task demands, Rakoczy and colleagues found that already three-year-old German children understand that people can have incompatible desires and may hence vary in how they evaluate similar problems [30]. Taken together, there is evidence suggesting that the capacity to understand and predict others’ choices based on their subjective preferences becomes fully consolidated by the age of 3 to 4 years among children growing up in Western, industrialized societies of the Global North.

Recent research has started to unravel cultural variation in young children’s social cognition. Wellman and Liu [34] found children from the US to master different mental state concepts in a developmentally consecutive sequence. These children first started to understand and predict others’ choices based on their desires before incorporating information on others’ beliefs and knowledge states. Children from Western, industrialized societies, such as the US, Germany, and Australia, almost uniformly attain these concepts in this fixed sequence [35–37]. In contrast, the development of Theory-of-Mind-related skills in children from some non-Western societies, such as China [35] and Iran [38], follows a different developmental sequence. Here, children readily utilize information on others’ knowledge states well before they consider others’ diverse beliefs.

While these studies suggest modest, gradual variation, other scholars have documented more prominent variation in children’s acquisition of Theory-of-Mind skills. For example, children from Samoa, an archipelago in the Pacific, have repeatedly been found to not ascribe false beliefs to others before reaching adolescence [39,40], but see [41], for different findings. Children from two other archipelagos in the Pacific, Vanuatu and Tonga, show similar late mastery of false belief understanding and other social-cognitive milestones [42,43]. Some researchers therefore suggested that this delayed emergence of a fully-fledged Theory of Mind may be explained in the light of culturally normative assumptions on the *opacity of mind doctrine* endemic to these societies in the Pacific region [e.g., 39,42,44]. Previously outlined in anthropological literature [44], this doctrine posits that speculations on what others think or feel are considered inappropriate in these societies and that such a doctrine shapes normative orders and everyday practice. Thus, as these may influence language use and empathy, but also Theory of Mind-related skills in interactions with others [44], the ’opacity of mind’ doctrine may discourage individuals from habitually making explicit inferences about what others think or feel, resulting in a delayed emergence of a fully-fledged Theory of Mind. Nevertheless, developmental researchers advise exercising caution regarding such a conclusion, due to the current absence of evidence supporting a causal relationship between the presence of the ’opacity of mind’ doctrine and a delayed onset of Theory of Mind-related skills. [39,42,45].

Alternatively, observed variation in children’s Theory of Mind across cultures may have been at least partly the result of methodological constraints: A common approach in cross-cultural research is that scholars adopt experimental procedures and paradigms originally developed and validated in Western societies to study other non-Western societies [46]. In such settings, an adult experimenter, who is unfamiliar to the participants, asks them about their beliefs, knowledge states, and desires of fictive characters, and participants need to verbally respond to these questions. While children in Western societies may be familiar with such dyadic, pedagogical settings, this may not be suitable in contexts in which children lack such experience. Indeed, using a more interactive false belief procedure, Callaghan and colleagues [41] found synchrony in the developmental onset of false belief understanding across 5 diverse societies, including Samoan children. Therefore, non-verbal tasks may represent a more suitable methodological approach to conduct more culture-fair studies of social-cognitive development across societies [41,47–50], as they avoid this primacy of mental-state talk typical for studies with children from Western societies [51] and the corresponding importance of language on Theory of Mind in these tasks [52].

Notably, a similar challenge applies to cross-species comparative research. Comparisons between humans and other primates are often difficult if not impossible to make when adopting language-based paradigms originally developed for research with humans to non-linguistic species. As a consequence, it remains unclear if species-level variation in social cognition at least partly results of these methodological adjustments [9]. However, some research indicates that apes, when facing inherently non-verbal study procedures, may show sophisticated socio-cognitive skills. For example, Buttelmann and colleagues introduced different ape species to a food-retrieval task [53]. After observing a human experimenter reacting very positively to one food type but uttering disgust to another, apes could pick one option following the experimenter’s secret choice. Interestingly, most apes chose the option that had been disliked by the experimenter, indicating a capacity for preference-based inferences among the apes [53]. However, since the apes may have simply reacted to the human’s emotional cues (e.g., the high-pitched voice in the ‘happy-condition’), it remains unclear whether apes, in the absence of such emotional cues, would have made similar inferences. In another study using a competitive food context, chimpanzees assumed others’ preferences to match their own [54]. When choosing between two food locations, chimpanzees chose against their preference only if a competitor had chosen before them, but not if they had the initial choice. However, the crucial benchmark for preference-based inferences, namely if apes understand that others can have preferences similar or different from their own, has not yet been tested.

In the present study, we combined a cross-cultural and cross-species comparison, employing consistent methods and measures across different cultural contexts and species. Our aim was to investigate whether the ability to comprehend the desires of other agents is exclusive to humans or shared with other species to gain deeper insights into the origins of human capacity for understanding the consequences of others’ preferences.

We compared human children from three diverse societies (Leipzig, Germany; Safotu/Samoa; Oshikoto region, Namibia) and four non-human great ape species to gain insight into the ontogenetic and phylogenetic roots of this fundamental capacity of social cognition. We assessed children from Leipzig, Germany, to validate our paradigm against previous work focusing on urban populations in Western, industrialized societies. We further tested children from Safotu, Western Samoa and ≠Akhoe Hai‖om children in northern Namibia. The Hai||om are traditionally semi-nomadic hunter-gatherers who parallel the Samoan children in their more limited access to e.g., Westernized technology and formalized schooling. Unlike for Samoa, there are no reports about cultural doctrines about others’ minds for the Hai||om or Germany. However, while it has been shown that 6-year-old Hai||om children ascribe diverse desires to fictive story characters, most children did not solve a verbal false-belief task [49]. However, Hai||om children at the same age readily mistrusted peer competitors while trusting peers with cooperative intent in a reward finding task [50]. This indicates that Hai||om children may tap more sophisticated socio-cognitive skills (i.e., anticipating deception/false beliefs) in behavioral, rather than verbal paradigms.

To enable comparisons with the nonhuman great apes, we employed a non-verbal response task, with only minimal verbal instructions for the human children. Participants could choose different food types depending on the previous food choice by one of two competitors. While participants shared the same preference with one of these competitors (*same preference* condition), their preference deviated from the second competitor (*opposite preference* condition). Competitors chose a food item first, while participants had no visual access to the scene. As such, participants had to infer which of the two items remained available based on preference-based inferences alone.

We predicted that if the participants would take their competitors’ preferences into account, their choices should differ depending on the experimental condition. In the *opposite preference* condition, they should choose their preferred food item as it would still be available, while in the *same preference* condition, participants should choose an alternative option since their preferred food might have been retrieved by their competitor. They could either choose their least preferred food still available on the table, or the ‘opt out’ option to which they had exclusive access. Since some previous studies suggest that children’s tendency to infer and act upon others’ mental states is shaped by the cultural context [39,42,43], we predicted systematic variation in the effect of condition across societies, with German children showing this capacity earlier than Samoan children. For the Hai||om children, we did not have specific predictions on whether their response patterns would align with either German or Samoan children. If the ’opacity of mind’ doctrine is crucial for shaping children’s performance in the current task, we would expect Hai||om children to resemble German children more than Samoan children. If, in contrast, exposure to Western formal education and conversation about mental states of others would be more relevant, we would expect these children to perform more similar to Samoan children. Alternatively, no substantial variation in the effect of condition across societies would indicate preference-based action anticipation as robust, early-emerging socio-cognitive skill in humans. For apes, we expected an effect of condition, such that participants would be more likely to choose their preferred food in the *opposite preference*, but not *same preference*-condition. No such effect would, in contrast, indicate that preference-based action anticipation is part of a human-specific socio-cognitive skillset. We did not have clear predictions of species-level variation in this skillset across ape species given the absence of prior research comparing these species and sample size limitations inherent to experimental research with apes. As such, such findings should be interpreted with caution in the absence of additional confirmatory tests.

## Materials and methods

### Sample

#### Human children

We tested a total of 71 children across three different societies. Samoan children were tested in Safotu, a small village on the northern coast of Savai’i, the larger of the two main islands comprising Western Samoa. From the approximately 40,000 inhabitants living on Savai’i, around 1,200 inhabitants live in the community of Safotu. The community had limited access to modern technology, and formal schooling was only recently introduced [55]. Twenty children from the Sacred Heart Primary School were recruited by opportunity sampling, comprising 12 boys and 8 girls (mean age: M = 6.65 years, SD = 1.27).

Leipzig is a city with approximately 600,000 inhabitants and is located in Germany, an industrialized Western European nation with mandatory formal schooling. Twenty-six children were selected from a database at the Max Planck Institute for Evolutionary Anthropology. This sample included 12 boys and 14 girls (mean age: M = 5.65, SD = 0.48).

The originally semi-nomadic ≠Akhoe Hai‖om have been resettled to Farm 6 in Mangetti West in northern Namibia and comprised a group of approximately 200 people at the time of data collection. In 2005, the |Khomxa Khoeda Primary School was built to introduce formal schooling to children, but attendance rates are low and irregular. Twenty-five children were recruited from this school using opportunity sampling, comprising 12 boys and 13 girls (age: M = 8.04, SD = 1.67).

Because both the Samoan and the ≠Akhoe Hai‖om children were recruited by opportunity sampling while the German children were recruited from a database, the children from the different cultural groups could not be fully matched regarding their age. While it was possible to recruit younger children in Germany and partly also in Samoa, the average age of the Namibian children was higher, as access to younger children was not possible.

#### Great apes

Data collection took place at Zoo Duisburg (gorillas, *Gorilla gorilla*), the Wolfgang Köhler Primate Research Center/Zoo Leipzig, Germany (bonobos, *Pan paniscus*; Sumatran orangutans (*Pongo abelii*) and at the Ngamba Island Chimpanzee Sanctuary, Uganda (chimpanzees, *Pan troglodytes*). We tested a total of 25 apes, including ten chimpanzees (seven males, three females), five bonobos (two males, three females), five gorillas (one male, four females), and five orangutans (all females). All participants lived with conspecifics in social groups of various sizes, with access to indoor and outdoor areas. Apes were tested in their indoor areas and fed according to their regular daily routine. Participants were not deprived of food or water at any time during testing.

### Ethics statement

#### Human children

The research was conducted in compliance with the ethical principles of the American Psychological Association (APA). The research with the German children as well as consent procedures were approved by an internal ethics committee of the Max Planck Institute for Evolutionary Anthropology, Leipzig. The “Working group for indigenous minorities in Southern Africa” (WIMSA) approved the research and consent procedures with the ≠Akhoe Hai‖om children in Namibia. The Ministry of Sports and Education in Apia, Samoa, granted permission to conduct the study in Samoa and reviewed the procedure accordingly. In Germany, parents gave their written informed consent. In Namibia, permission to recruit children and to conduct the study at the local school was given by its headmaster. Before the research started, he obtained verbal consent from the children’s parents. Written consent was not feasible because of illiteracy among the Hai‖om. In addition, In Samoa, verbal consent was given by the headmaster of the school, who was in a position of parental authority. In both Namibia and Samoa, before testing, the children watched a video clip with the instructions for the study presented in their mother tongue, after which they were asked individually for their verbal consent, which was documented on video. Only if they gave their consent, data collection started (see also S4 File).

#### Great apes

Research for the apes in Leipzig was approved by an internal ethics committee at the Max Planck Institute for Evolutionary Anthropology, consisting of scientists, zookeepers and a veterinarian. An internal ethics committee approved the research for the apes in Duisburg Zoo at the Zoo Duisburg consisting of zookeepers and the curator for the apes. The great apes in both locations live in semi-natural indoor and outdoor enclosures, containing climbing structures such as trees, ropes, platforms, and a variety of permanently installed environmental enrichment devices. This feeding and enrichment routine did not change throughout testing. Research for the chimpanzees on Ngamba island was approved and reviewed by the Ugandan Wildlife Authorities (UWA), the Ugandan National Council for Science and Technology (UNCST), and the Chimpanzee Sanctuary & Wildlife Conservation Trust. Participants voluntarily participated and were never deprived of food or water. Research complies with the “Guidelines for the Treatment of Animals in Behavioral Research and Teaching” of the Association for the Study of Animal Behavior [56].

### Materials and setting

#### Human children

**Fig 1:**
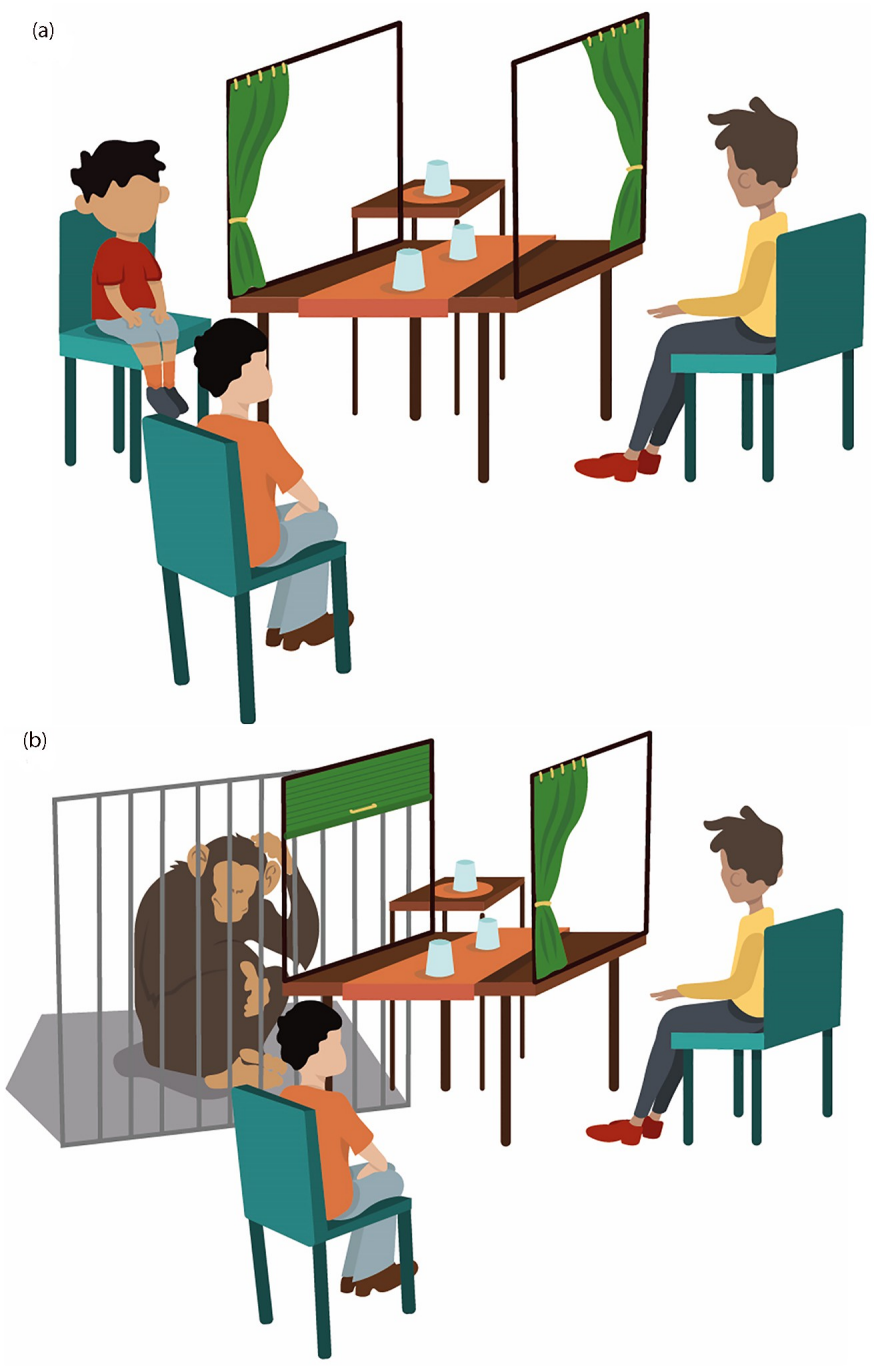
General setting for the human children (a) and for the apes (b). The setting shows the experimenter (orange shirt) sitting between the participant (on the left) and the competitor (yellow shirt, on the right) with the cups between the participant and the competitor and the ‘opt out’ option next to the participant.

Three chairs were placed on three sides of a table for the participant, the competitor, and the experimenter to sit on. The child sat opposite to an adult competitor, who acted as a research confederate. Between them was a table on the top of which a sliding board (61 cm x 19.5 cm) was mounted. Two opaque cups (diameter = 7.4 cm, height = 9.4 cm) were placed on the board. An identical third cup was placed on a table next to the child to which only they had access (‘opt out’ option). Participants indicated their choice by pointing to the cup they wanted the experimenter to lift. We attached a frame with curtains to the table which the experimenter could close to block the child’s or the competitor’s view (see Fig 1a). Food rewards varied across societies to ensure that children were familiar with the options and that food was locally available (see *Preference Assessment Phase*).

#### Great apes

A similar setting was used for the apes (Fig 1b). Two stools were placed on two sides of the table for the human competitor and the experimenter to sit on. A table was placed between the participant and the human competitor with a sliding board (80 cm x 12 cm) attached to it. A third cup was placed on a table next to the participant, representing the ‘opt out’ option to which only the participant had access. Apes indicated their choice by pointing through holes in a Plexiglas panel or through metal bars to the cup they wanted the experimenter to lift. A choice was only considered made, if only one cup was indicated at a given time. Attached to the table was a frame (75 cm x 50 cm x 52 cm) with PVC occluders (75 cm x 50 cm), which the experimenter could close to block the participant’s or the competitor’s view. Food rewards varied across species depending on the species’ preferences and availability (see below).

### General procedure

#### Preference assessment phase

Three food options were introduced to participants to assess their preferences. This included the participants’ most preferred option, the participants’ least preferred option, and an intermediate option (i.e., ‘opt out’ option). The ‘opt out’ option was introduced to grant access to food in situations during which their most preferred option was most likely gone.

To establish individual food preferences, each participant received 12 trials of a food-preference test to rank their *most preferred* over *intermediate* to *least preferred* food options. For children, food options were sweets or chocolate, which were preferred by most children, pretzels or nuts as the intermediate option as indicated by most children, and dried tomatoes as the least preferred food. Apes’ food options were bananas or pasta as the most preferred option, carrots or cucumber as the intermediate option, and eggplant or radish as the least preferred option. Food types were presented in pairs of two in all possible combinations, and the participants’ preferences within each combination were noted. A food item was considered the preferred option if it was chosen at least five out of the six times it was paired with another food type. The least preferred food had not to be selected more than once. After each individual’s food preference was established, participants entered the experimental phase.

#### Experimental phase—Manipulation

The experimental phase included two conditions, which were assessed in separate sessions following a within-participant design. Sessions were presented on two consecutive days. Participants were confronted with one of two different adult human competitors. One competitor demonstrated a food preference identical to the participant’s preference (*same preference* condition). If, for example, the participants’ most preferred food was chocolate, the competitor would also demonstrate a preference for chocolate. The other competitor demonstrated the opposite preference (*opposite preference* condition), opting for the participant’s food type least preferred. Thus, if the participant’s least preferred food were dried tomatoes, the competitor would demonstrate a preference for dried tomatoes.

Participants received one session per condition. Each session started with a demonstration of the respective competitor’s preference. The experimenter placed two transparent cups to demonstrate the competitor’s preference, each filled with one of the two food types (the participant’s *most preferred* food and the participant’s *least preferred* food) on the sliding table between participant and competitor. Next, the experimenter slid the table to the competitor’s side. The competitor indicated their choice by clearly pointing to the corresponding cup and reaching for the respective food. The experimenter then removed one piece of food from this cup and handed it to the competitor. The competitor demonstrated satisfaction upon receiving the food by showing positive facial expressions (e.g., smiles for human children) and appropriate sounds (e.g., “hmmm” for human children and food grunts for the apes). The competitor collected the food for later consumption with the children, and the children were also instructed to do so. In the case of the apes, the competitor pretended to eat the food right away. After the competitor had demonstrated their preference in six consecutive trials, the experimental phase began.

#### Experimental phase—Test

Opaque cups were baited such that the *most preferred* and the *least preferred* option were present on the sliding table. In contrast, the *intermediate* option (‘opt out’) was presented on the separate table next to the participant, to which the latter had exclusive access. The experimenter baited all three cups with one piece of the respective food option in full view of both competing individuals. The experimenter started with the intermediate option on the table to the participant’s side before continuing with the two cups on the sliding table. The one closer to the experimenter was always baited first. After all cups were baited, the participant’s view was occluded so that they could not see the competitor’s choice. The competitor chose by pointing to the cup she wanted the experimenter to lift. After the competitor had chosen and had received her reward, the experimenter removed the occluder and slid the table toward the participant for them to choose. To ensure the participant’s motivation during the session, they also received ‘motivation-trials’. Here, the participant could choose first (unseen by the competitor), before the table was moved to the competitor’s side. Motivation trials were semi-randomly interspersed, with the stipulation that they could not be the first trials the participant experienced and that two of them could not be conducted consecutively. Motivational trials were not included in later analyses. Participants received eight experimental trials and two motivation trials in each session and one session per condition.

The location of the most preferred and the least preferred option were counterbalanced across trials and semi-randomized, with the restriction that an option could never be in the same location for more than two consecutive trials. After the first session (i.e., test of first condition) was finished, the competitor changed, and the participant engaged in the remaining condition. Half of the participants started with the *same preference* condition, and the other half started with the *opposite preference* condition.

All trials were video-recorded, and participants’ choices (cup with most preferred food; cup with least preferred food, opt out option with neutral food) were later coded from video by AG. To establish reliability for which cup was chosen by the subject (most preferred, least preferred, intermediate/opt out option), a second coder coded 20% of the data (380 trials). Agreement between coders was 98%, with a Cohen’s kappa of 0.91, indicating a very high level of agreement between the two coders beyond what would be expected by chance.

### Data analyses

We fitted generalized linear mixed models with Bernoulli response distributions in *R* [57] using *brms* [58,59] to assess whether participants considered their competitor’s preferences (*condition*: same; opposite) when deciding which option to choose (*preferred choice*: preferred food option; non-preferred food option). Furthermore, we assessed whether the effect of condition would vary across human *societies* (child data: Germany; Namibia; Samoa) or across non-human great ape *species* (ape data: chimpanzee; bonobos; orangutan; gorilla). Models were fitted using the *brm* function using default (weakly informative) priors. Model formula were similar for child data and ape data to allow for ideal comparability of results. Data and codes are available as (S2 and S3 Files).

For each dataset, we compared the predictive accuracy of four different models to assess the effects of the two predictors on the response (*preferred choice*). We utilized widely applicable information criterion (WAIC) and weights [60, see 61 for a similar approach]. A model’s WAIC informs about the predictive accuracy of the model, with lower scores indicating better accuracy. WAIC weights estimate the probability that a model will make the best predictions regarding new data in relation to the competing models. Following McElreath [60], we report WAICs together with their standard errors alongside with the WAIC weights to nominate models producing the best predictions while also considering their parsimony. To further illustrate the effects of the predictors, we also provide the estimates and the 95% highest posterior density (HPD) intervals for the full models comprising the main effects and the potential interactions between predictors.

Running the full models (M_Full Children_; M_Full Apes_), we fitted fixed effects of the predictors *condition* (same, opposite) and *society* (human children; statistical analysis 1) or *species* (great apes; statistical analysis 2) as well as their two-way interaction. We further added information on participants’ *age* (scaled) as a control variable. Further, we added random intercepts of participants’ *IDs* to account for the repeated measures design of the study. We also added random slopes of *trial* (scaled) per *ID* to account for order effects in the study.

First, we compared these full models to null models lacking the two predictors and their two-way interactions. To further investigate whether potential effects of *condition* would vary across *societies* or *species*, we then compared the predictive accuracies of the full models to reduced (i.e., main effects only) models lacking the interaction between the two predictors. Finally, we compared the predictive accuracy of the main effects only model with reduced models lacking information about either *condition* or *society*/*species* to test potential main effects of these predictors.

On a descriptive level, we also inspected participants’ ‘opt out’ choices to assess whether this strategy was used selectively when facing competitors with similar food preferences. In an explorative analysis, we also ran inferential analyses following the same logic as above to predict participants ‘opt out’ choices by their *condition* and *society*/*species*. These analyses were, however, largely redundant to the main analysis since choosing the preferred food option implied the refusal of the remaining options (and vice versa). Moreover, we encountered complete separation issues in this analytic step for some models (i.e., none of the orangutans chose the ‘opt out’ option, resulting in convergence issues). We thus decided to add these analyses to the (S1 File).

## Results

### Human children

WAIC weights suggested that the full model (M_Full Children_: *WAIC* (*SE*) = 1130.9 (35.8); *weight* > .999) predicted children’s *preferred choices* better than the null model (M_Null Children_: *WAIC* (*SE*) = 1391.7 (23.7); *weight* < .001). Comparing the full model to a more parsimonious main effects only-model indicated no clear support for the former (*weight* = .561) over the latter (M_Condition*Society Children_: *WAIC* (*SE*) = 1131.4 (35.5); *weight* = .439). Comparing the main effects only model (*weight* = .435) to a model lacking *condition* (M_Condition Children_: *WAIC* (*SE*) = 1385.3 (24.9); *weight* < .001) and a model lacking *society* (M_Society Children_: *WAIC* (*SE*) = 1130.9 (35.1); *weight* = .565) showed a strong effect of *condition* on children’s *preferred choices*. These model weights indicated no clear effect of *society* on children’s *preferred choices* (i.e., similar weights for both models), but also no clear evidence for the absence of such an effect. In this case, we would prioritize the more parsimonious M_Society Children_ and emphasize that children across all societies took into account their competitors’ preferences.

**Fig 2:**
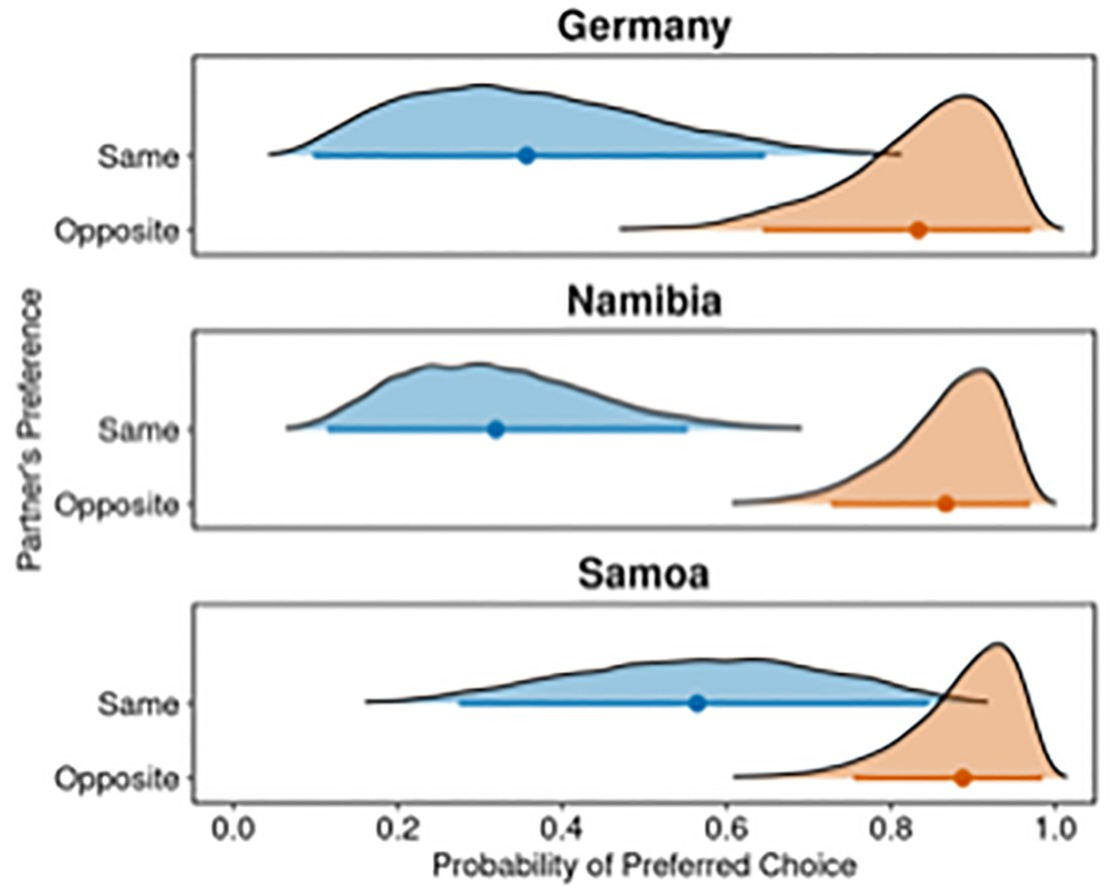
Posterior density plots estimating the probabilities that a child will choose their preferred food options according to the full model (M_Full Children_). Distributions are presented separately for each condition and society. Children’s ages and trial number are set at 0. Dots present posterior means, horizontal lines 95%- HPD intervals.

In sum, children across societies refrained from choosing their preferred option if their competitor had shown the same food preferences as them. There remains some uncertainty as to whether this capacity varied to some extent across societies, but our analyses indicate that such an effect would, if present, be subtle. Observation of the 95%-HPD intervals per condition and society further illustrates this interpretation (Fig 2): Samoan children showed a slightly higher variability in their choices when facing a competitor with similar food preferences than German and Namibian children. However, children from all three societies considered their competitor’s food preferences when choosing after them.

On a descriptive level, children’s ‘opt out’ choices indicated that children across societies used this strategy to safeguard against loss in the same preference condition (prob_Germany_ = .80; prob_Namibia_ = .65; prob_Samoa_ = .60), but less so in the opposite preference condition (prob_Germany_ = .33; prob_Namibia_ = .30; prob_Samoa_ = .18).

### Great apes

Inspection of WAIC weights indicated that the full model (M_Full Apes_: *WAIC* (*SE*) = 436.0 (28.7); *weight* = .829) outperformed the null model (M_Null Apes_: *WAIC* (*SE*) = 439.2 (25.9); *weight* = .171), although this difference in predictive accuracy was rather small. Comparing the full model (*weight* = .862) to a main effects only model (M _Condition*Species Apes_: *WAIC* (*SE*) = 439.7 (26.9); *weight* = .138) indicated that an interaction between *condition* and *species* may cause this effect. Indeed, comparisons between the main effects only model (*weight* = .264) and models lacking *condition* (M_Condition Apes_: *WAIC* (*SE*) = 438.4 (26.8); *weight* = .501) or *species* (M_Species Apes_: *WAIC* (*SE*) = 440.0 (26.4); *weight* = .231) indicated no support for any main effects. As such, the full model had slightly better predictive accuracy than the most parsimonious null model.

**Fig 3:**
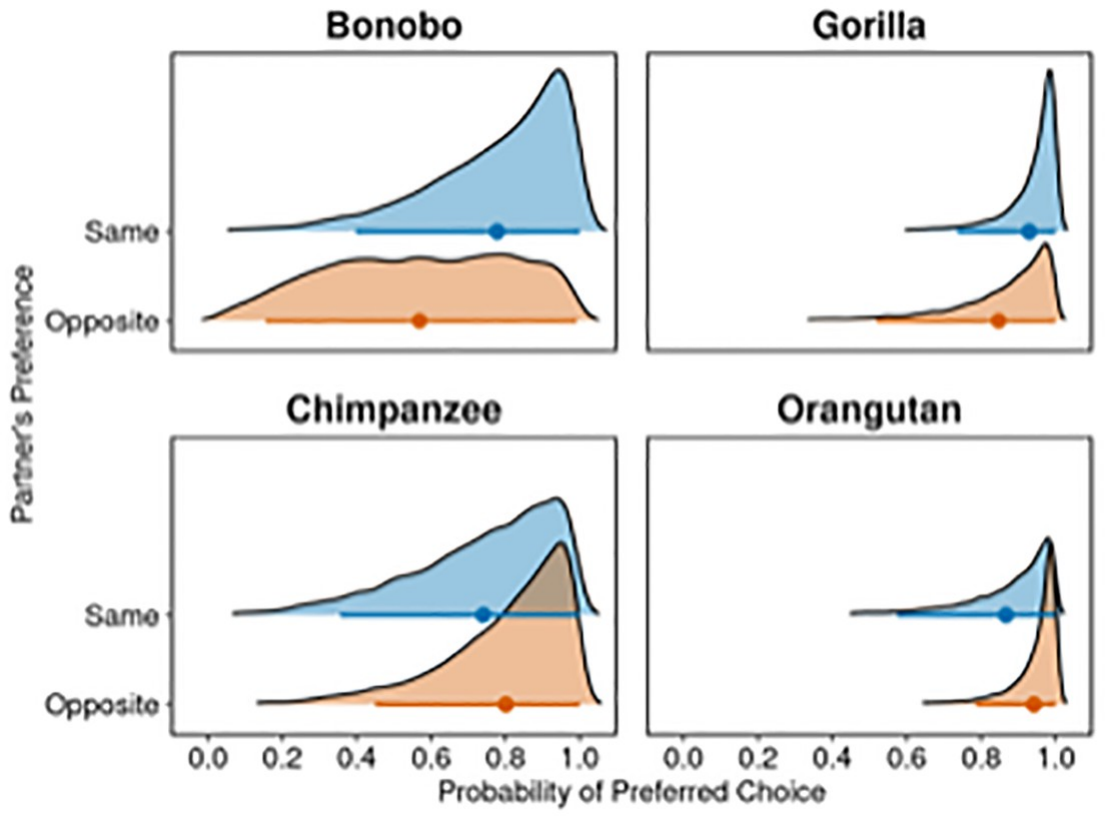
Posterior densities regarding the probabilities that an ape will choose it’s preferred food options according to the full model (M_Full Apes_). Distributions are given separately for each condition and species. Apes’ age and trial number are set at 0. Dots present posterior means, horizontal lines 95%- HPD intervals.

Observation of the 95%-HPD intervals per condition and species indicates some species-level variation in apes’ choices (Fig 3). While all four species uniformly chose their preferred food options across condition, bonobos were more likely than other species to also consider alternative options. Importantly, they did not do so selectively when facing a competitor with similar food preferences but did so regardless of their competitors’ food preferences.

The descriptive analysis of bonobos’ food choices indicated that this effect was mainly driven by a preference for the ‘opt-out’ option among some individuals (see also S1 File). More specifically, bonobos were more likely to choose this option than other non-human great ape species, and this effect was particularly pronounced for the same preference condition (prob_Bonobo_ = .30; prob_Chimpanzee_ = .04; prob_Gorilla_ = .06; prob_Orangutan_ = .00), but, to a lesser extent, also for the opposite preference condition (prob_Bonobo_ = .14; prob_Chimpanzee_ = .12; prob_Gorilla_ = .02; prob_Orangutan_ = .00).

In sum, our results indicate substantial variation in apes’ food choices across species and condition. However, unlike humans, none of the four great ape species showed a clear strategy of choosing their preferred options only when facing competitors with opposing preferences while choosing other food options when facing competitors with similar preferences. Instead, all four species chose their preferred options in most of the trials and did so regardless of condition.

## Discussion

By combining cross-cultural and cross-species comparisons, we investigated whether human children from three different cultural backgrounds and several species of non-human great apes consider the others’ preferences even if those might be different from their own. By applying this rarely used approach, we could show that in all three human societies, children at 5 years of age and older consider their competitor’s preferences in a competitive food-choice task, while we found no evidence for such a capacity among any of the four great ape species. In more detail, children from all three societies avoided their preferred food options (and typically opted out by choosing the food option only they had access to) when their competitor’s preference was identical to theirs. If preferences were dissimilar, children from all three societies went for their preferred food. It is important to note that, when demonstrating their preferences, the competitors did not express any emotions towards the food they were eating. This means that simple association with negative or positive expressions cannot explain the findings. Our results therefore show that children understood that another individual’s preferences can be different (or identical) to their own and acted in accordance.

While our study confirmed findings of previous research with German, even younger children [30], we additional show the presence of this skill in two other, non-Western societies. Thus, our expectation that Samoan children might differ from German and Hai‖om children was not confirmed, as preference-based reasoning was present in children from all three societies. Our findings therefore seem to suggest that for preference-based reasoning, like for other Theory-of-Mind related skills, cultural background seems to have little influence on its emergence in ontogeny [34,41,62]. There are, however, several explanations for this finding. First, it could be argued that the children in the current study were too old to capture the onset of preference-based reasoning, to be able to detect variability across societies. This is supported by recent studies from Pacific small-scale societies [42,43], showing that both rural ni-Vanuatu and Tongan children did pass the ‘diverse desire’-task, administered as part of a Theory-of-Mind Scaling test, already at similar ages as the Samoan children tested in the current study. However, both studies also found that ni-Vanuatu and Tongan children showed a delayed onset in mastering false belief task. Thus, most urban ni-Vanuatu children passed the task after 7 years, while the majority children from rural areas only passed after 9 years [42]. The majority of Tongan children passed two different false belief tasks at the ages of 6 and 7 years, respectively [43]. Although we did not consider additional Theory of Mind-skills other than understanding others’ preferences, this highlights the importance of studies investigating the onset and order of emergence of several Theory-of-Mind related skills, as these patterns might vary across cultural contexts [38,42,43,45]. Finally, our findings might be explained by our use of an interactive, less language-based experimentally setting, as it has been demonstrated that more behavior-based approaches are less likely to find a delayed onset or absence of Theory of Mind-related skills in Samoan children [41].

Rakoczy and colleagues [30] suggested for three-year-old German children, incompatible desires are easier to understand than compatible ones. We could not confirm this finding, as children chose strategically in both conditions, taking their competitor’s corresponding preference into account. This discrepancy between studies might be explained by the fact that we tested older children, who were better able to clearly differentiate between their own and others’ preferences. Furthermore, our less language-based setting could have been easier to understand than the more verbal setting of Rakoczy et al. [30]. Furthermore, they have used puppets, acting in different scenarios and children were then asked them to answer questions regarding to the puppets’ desires and emotions. This, however, requires the ability of symbolic representations of people, which has been demonstrated to follow diverse developmental trajectories across societies [63].

Unlike human children, great apes did not take their competitor’s preferences into account when choosing from different food options. It is unlikely that the setting was too difficult for the apes, as similar paradigms have already been successfully applied to investigate other, Theory-of-Mind related skills [4,54]. It is also unlikely that the apes found the ‘opt-out option’ too difficult to understand, as the pre-training showed that they opted out immediately if they had seen the other individual choose their preferred food. Moreover, results indicated ape species opted out in at least some trials. Interestingly, bonobos seemed to opt out more often than the other species, suggesting that bonobos could be generally more risk averse and therefore prefer the “fixed” (and therefore safe) option [64].

In summary, our results show that unlike human children from different societies, great apes did not take their competitors’ preference into account when making their choice. This seems to suggest that understanding others’ preferences, at least with regard to food, is limited to humans, where it is present in middle childhood, most likely largely independent from the cultural context.

However, this study suffers from some weaknesses. First and foremost, our samples sizes were small, and the mean ages of children varied across societies, which was due to the limited access to sufficient numbers of human participant, particularly in Namibia, but also to sufficient numbers of apes. It is possible that with larger sample sizes, we might have detected subtle differences between conditions, societies or species. It is also possible that for the children, competing against an adult competitor introduced a level of pressure, which might have affected their overall choices. However, we think that this potential power asymmetry would have only increased the need to come up with a successful strategy.

One could also argue that for the apes, having a human competitor induced a mismatch in the understanding of food preferences. Consequently, this may have resulted in a disadvantage for the apes, as they did not interact with a conspecific and for example, could have paid more attention to a conspecific as a competitor. However, there is a series of studies showing that chimpanzees understand and respond to human competitors [4,54,65], similarly as they do to a conspecific competitor [5,66]. It is further possible that the task was cognitively too challenging for the apes, as it required them, at least in some situations, to inhibit their own preference for their preferred food in favor of a strategically more appropriate choice. This is supported by previous work in which chimpanzees struggled to inhibit their initial responses in a setting where pointing to a lower quantity led to the bigger reward [67]. However, Rosati et al. [68] showed that in a delayed gratification test, bonobos and, to an even greater extent, chimpanzees, were able to wait patiently in front of a reward, inhibiting their response while waiting for the bigger reward to come. In addition, Kaminski et al. [4] demonstrated that chimpanzees manage to inhibit their efforts to obtain a higher quality reward in situations where, based on their understanding of the situation, it was most likely gone.

## Supporting information

S1 File Supporting information. (DOCX)

S2 File Data children analysis. (CSV)

S3 File Data ape analysis. (CSV)

S4 File PLOS questionnaire on inclusivity. (DOCX)
