# Supplementary material for: Understanding others’ preferences: A comparison across primate species and human societies
Source: PLoS One. 2024 Jan 17;19(1):e0295221. doi: 10.1371/journal.pone.0295221 (PMC10793897; doi:10.1371/journal.pone.0295221)
Supplement: S1 File — (DOCX) [file pone.0295221.s001.docx]

**Supplementary Materials**

In an explorative analysis, suggested by a reviewer, we predicted participants choices of the ‘opt out’ option following a similar approach as the one outlined in the main manuscript. It is important to note that for some of the analytic steps addressing effects of *species* (Ape Data) indicated convergence issues. These were most likely the result of complete separation issues given that none of the Orang-Utan subjects chose the ‘opt out’ option throughout the study. As such, these results should be treated with caution. We report the descriptive data regarding participants ‘opt out’ choices in the manuscript.

*Human Children*

The full model (M_Full Children_: *WAIC* (*SE*) = 847.6 (42.1); *weight* > .999) predicted children’s *‘opt out’ choices* better than the null model (M_Null Children_: *WAIC* (*SE*) = 1189.6 (29.6); *weight* < .001). The full model (*weight* = .520) did not predict novel data substantially better than the more parsimonious main effects only model (M_~~Condition*Society~~ Children_: *WAIC* (*SE*) = 847.7 (41.1); *weight* = .480). Finally, the main effects only model (*weight* = .758) had much better predictive accuracy than a model lacking *condition* (M_~~Condition~~ Children_: *WAIC* (*SE*) = 1186.4 (29.7); *weight* < .001). A model lacking *society* (M_~~Society~~ Children_: *WAIC* (*SE*) = 850.0 (41.1); *weight* = .242) had marginally lower predictive accuracy in comparison.

In sum, children across societies chose the ‘opt out’ option selectively when their competitor had shown similar food preferences than themselves. There remains some uncertainty whether this tendency differed across societies, but such effects would be markedly small.

*Great Apes*

The full model (M_Full Apes_: *WAIC* (*SE*) = 196.6 (26.4); *weight* = .91) showed better predictive accuracy than the null model (M_Null Apes_: *WAIC* (*SE*) = 201.4 (23.1); *weight* = .087). The full model (*weight* = .973) also outperformed the main effects only model (M _~~Condition*Species~~ Apes_: *WAIC* (*SE*) = 203.8 (24.4); *weight* = .027) suggesting that an interaction between *condition* and *species* may be of relevance in predicting ‘opt out’ choices. Further analyses comparing the main effects only model (*weight* = .196) and those lacking *condition* (M_~~Condition~~ Apes_: *WAIC* (*SE*) = 201.2 (24.1); *weight* = .713) and *species* (M_~~Species~~ Apes_: *WAIC* (*SE*) = 205.3 (24.6); *weight* = .091) suggested some support for the notion that *species* would vary in their ‘opt out’ choices.

More specifically, our data suggested that Bonobos tended to choose the ‘opt out’ option more often than the other Great Ape species, who did so very rarely (see descriptive data in main manuscript). Thus, only Bonobos show some variation in their ‘opt out’ preferences across conditions. It is important to note, however, that they appeared to do so more often when their preferences were opposed to those of their partner.
