# Supplementary material for: Understanding others’ preferences: A comparison across primate species and human societies
Source: PLoS One. 2024 Jan 17;19(1):e0295221. doi: 10.1371/journal.pone.0295221 (PMC10793897; doi:10.1371/journal.pone.0295221)
Supplement: S4 File — (DOCX) [file pone.0295221.s004.docx]

Inclusivity in global research

PLOS’ policy on inclusivity in global research aims to improve transparency in the reporting of research performed outside of researchers’ own country or community and ensures that PLOS publications reporting global research adhere to high standards for research ethics and authorship. Authors of relevant research articles may be asked to complete the questionnaire below, which outlines ethical, cultural, and scientific considerations specific to inclusivity in global research. This questionnaire may be requested when researchers have travelled to a different country to conduct research, if research uses samples collected in another country, research with Indigenous populations or their lands, or if research is on cultural artefacts. Researchers travelling to another country solely to use laboratory equipment will not normally be required to complete the questionnaire. However, the questionnaire can be requested at the journal’s discretion for any submission – if you have been requested to complete this questionnaire by the PLOS journal you submitted to, please do so.

Please complete the questionnaire below and include this as a Supporting Information file with your manuscript. Note that if your paper is accepted for publication, this checklist will be published with your article in the supporting information files. Please ensure that you reference the checklist in the main body of your manuscript. We suggest adding a subsection ‘Inclusivity in global research’ to your Methods section and adding the following sentence: “Additional information regarding the ethical, cultural, and scientific considerations specific to inclusivity in global research is included in the Supporting Information (SX Checklist)”

The questions have been designed to be applicable to a wide range of study types, and there are subsections for both human subjects research and non-human subjects research. If any of the questions are not relevant to your research please mark them as “N/A” as appropriate.

**Ethical considerations, permits and authorship**

*This section is applicable to all research types.*

Provide details as to who granted permissions and/or consent for the study to take place in the Methods section of your manuscript. This should include the names of **all** ethics boards, governmental organizations, community leaders or other bodies that provided approval for the study. If individuals provided approval refer to these people by their role or title but do not list their name(s).

Reported on page number: page 11

If there were any deviations from the study protocol after approval was obtained please provide details of these changes in the Methods section of your manuscript.

Reported on page number: NA

Did this study involve local collaborators that are residents of the country where the research was conducted or members of the community studied? If you do not have any authors from said communities, please provide an explanation for this below.

The principal of the|Khomxa Khoeda Primary School, Ephraim Kavetuna, who is not a member of the ≠Akhoe Hai‖om, gave permission to conduct the study at the school. Disney Tjizao, who is also not a member of the community, but previously a teacher at this school, translated the instructions into Hai‖om and videorecorded them for us. According to the PLOS policies regarding authorship, this does not sufficient to justify authorship. Both are named in the acknowledgements.

Everyone listed as an author should meet PLOS’ criteria for authorship and all individuals who meet these criteria should be included in the author byline, rather than the acknowledgements. Authorship criteria is based on the International Committee of Medical Journal Editors (ICMJE) Uniform Requirements for Manuscripts Submitted to Biomedical Journals - for further information please see here: <https://journals.plos.org/plosone/s/authorship>.

**Human subjects research (e.g. health research, medical research, cross-cultural psychology)**

Did you obtain written informed consent from a representative of the local community or region before the research took place? How did you establish who speaks for the community? Details of written informed consent obtained from study participants should be reported separately in the Methods section of your manuscript.

Page 11: The “Working group for indigenous minorities in Southern Africa” (WIMSA) approved the research and consent procedures with the ≠Akhoe Hai‖om children in Namibia. The Ministry of Sports and Education in Apia, Samoa, granted permission to conduct the study in Samoa and reviewed the procedure accordingly. In Germany, parents gave their written informed consent. In Namibia, permission to recruit children and to conduct the study at the local school was given by its headmaster. Before the research started, he obtained verbal consent from the children's parents. Written consent was not feasible because of illiteracy among the Hai‖om. In addition, In Samoa, verbal consent was given by the headmaster of the school, who was in a position of parental authority. In both Namibia and Samoa, before testing, the children watched a video clip with the instructions for the study presented in their mother tongue, after which they were asked individually for their verbal consent, which was documented on video. Only if they gave their consent, data collection started.

How did members of the local community provide input on the aims of the research investigation, its methodology, and its anticipated outcome(s)?

We received input from the school principals and teachers in both Samoa and Namibia, when we first presented the aims and intended procedures of this study, e.g., if it would be more appropriate to play this game with a stranger (researcher) or a community member (teacher). Several people (Namibia: Christian Khamkhaeb, Disney Tjizao (teacher, different community); Samoa: Sister Maria Tevaga, Sister Camelia) provided their input when translating/backtranslating the instructions, regarding specific phrases to be used or how to express the instructions in a child-appropriate way.

When engaging with the local community, how did you ensure that the informed consent documents and other materials could be understood by local stakeholders?

In Namibia, we were supported by a linguist, Dr. Christian Rapold, who was working with this community for many years, documenting their language, with translating the instructions into the local language. In addition, the teacher Disney Tjizao and a member of the local community, Christian Khamkhaeb, translated and back-translated the instructions to make sure they were understood by the ≠Akhoe Hai‖om. In Samoa, two Sister Maria Tevaga, Sister Camelia ensured that informed consent documents were understood by local stakeholders.

Will the findings of the research be made available in an understandable format to stakeholders in the community where the study was conducted (e.g. via a presentation, summary report, copies of publications, etc.)? Please provide details of how this will be achieved.

The results of the study will be presented to the members of both communities. In Namibia, this will be done during our next research visit in the form of a short presentation (translated in to Hai‖om). A copy of the publication will also be sent to WIMSA. In Samoa, the publication will be circulated via email to the representative of the local school.

**Non-human subjects research using specimens/ animals collected as part of the study, or those housed in archival collections. Examples include archaeology, paleontology, botany and zoology.**

Did the permission you obtained from a local authority to perform the study include an agreement on access to outputs and benefit sharing? This may include procedures to enable fair distribution of the benefits and resources arising from the research performed. Please include any details of Prior Informed Consent and Benefit Sharing Agreements obtained. These may be required by field-specific regulations, for example the Convention on Biological Diversity (CBD) and the associated Nagoya Protocol.

N/A

If the material used in your study was imported, please A) provide the year it was imported and B) indicate whether permits were obtained to import/export the materials used, C) provide details of any permits obtained. If this information is not available, please indicate this.

N/A

If you used archival specimens, please state how the material used in your study was acquired by the institute it is held in and provide details of any permits obtained for the original excavations/ sample collection. If this information is not available, please indicate this.

N/A

How was the potential cultural significance of the materials collected in your study to local communities considered in your research design? Were Indigenous peoples and/or local researchers and institutions involved with archaeological excavations / collection of specimens? If so, please provide a description of their involvement.

N/A

If your manuscript includes photographs of human remains please indicate whether authors obtained permission from descendants or affiliated cultural communities to do so.

N/A
